# Editorial: Aging, peripheral inflammation, and neurodegeneration

**DOI:** 10.3389/fnagi.2024.1529026

**Published:** 2024-12-10

**Authors:** Caroline Haikal, Robert Weissert

**Affiliations:** ^1^Neurological Surgery, Weill Cornell Medical Center, New York-Presbyterian, New York, NY, United States; ^2^Department of Neurology, University of Regensburg Hospital, Regensburg, Germany

**Keywords:** aging, amyotrophic lateral sclerosis, neurodegenerative diseases, neuroinflammation, inflammation, Parkinson's disease, Alzheimer's disease, oxidative stress

## 1 Editorial overview

Aging is the major risk factor for a multitude of diseases including neurodegenerative diseases such as Parkinson's disease (PD) and Alzheimer's disease (AD). Understanding aging-related biological changes is crucial to identifying why some individuals develop neurodegenerative diseases while others do not. Aging is a heterogeneous process, both in regard to individuals within a population, and within tissues and organs affected in an individual. As such, considerable effort has been put toward understanding the biological age of systems as opposed to relying on chronological age. Biological aging is characterized by 12 hallmarks (López-Otín et al., 2023), many of which are linked to neurodegenerative disease mechanisms. However, many of these hallmarks have bidirectional relationships with each other. Indeed, a recent review highlights the bidirectional relationship of chronic inflammation with each of the other 11 hallmarks (Baechle et al., 2023). An integrated view of aging and inflammation could aid in bettering the understanding of neurodegenerative diseases and identifying new avenues of research and effective therapeutic strategies. We have arranged this Research Topic into three broad subjects, as follows: (A) Oxidative stress and metabolic dysregulation in neurodegenerative diseases; (B) Immune system and inflammatory mechanisms in neurodegenerative and systemic diseases; and (C) Genetics, aging, and systemic conditions related to neurodegenerative risk.

## 2 Oxidative stress and metabolic dysregulation in neurodegenerative diseases

A recent study examined the directional relationships in which the biological age of one organ selectively affects the biological age of other organ systems and found that the advanced age of several body systems explained advanced brain age (Tian et al., 2023). In the review by Kong et al., the authors explore how oxidative stress links brain and lung health. Oxidative stress has damaging effects on lipids, proteins and DNA all of which are affected in pulmonary diseases, such as chronic obstructive pulmonary disease (COPD) and fibrosis as well as brain diseases such as stroke, traumatic brain injury (TBI), and neurodegenerative diseases. They also examine the role of reactive oxygen species (ROS) in brain-lung crosstalk, identifying amongst other things, iron homeostasis disruption, breakdown of the blood brain barrier, and activation of the immune system. In their bibliometric analysis, Liu et al. map the research landscape of ferroptosis, an iron-dependent form of cell death, within neurodegenerative diseases. The study shows that ferroptosis research, particularly related to AD and PD, has grown significantly. This cell death pathway is associated with oxidative stress and lipid peroxidation, suggesting it may be a target for therapeutic intervention across neurodegenerative diseases. The role of iron dysregulation in neuroinflammation is further examined by Ashraf et al.. In this study, the authors examined the effects of lipopolysaccharide (LPS)-induced inflammation on cognitive performance and regional brain metabolism following mild iron priming. They reported that iron priming had different metabolic effects on the hippocampus and cortex but induced hyperramification of microglia in both regions following LPS administration. The nuanced, region-specific brain responses to iron priming-both alone and when combined with LPS-induced inflammation underscore the need for comprehensive analyses in such studies, and reinforce a link between iron dysregulation, inflammation, and neurodegenerative disease. Perturbation of iron (and copper) homeostasis is also discussed in Wimalasena et al.'s article. Here, the authors propose a new hypothesis to account for the selective vulnerability of neuromelanin-containing dopaminergic neurons in the substantia nigra pars compacta and noradrenergic neurons in the locus coeruleus. They argue that these neurons' high energy demands and complex metabolic roles render them more susceptible to oxidative stress and metal dysregulation (particularly iron and copper), which, in turn, promotes alpha-synuclein aggregation. This aggregation is a critical factor in Parkinson's disease pathology, providing a comprehensive framework for understanding selective neurodegeneration.

## 3 Immune system and inflammatory mechanisms in neurodegenerative and systemic diseases

Kitchener et al. studied a lysosomal enzyme, beta-galactosidase, the overexpression of which has previously been implicated in AD. Using immortalized BV-2 cells and primary mixed glial-neuronal cultures, as well as *in vivo* studies of young and aged mice, they show an upregulation of microglial beta-galactosidase cell-surface activity and extracellular release in response to inflammation which in turn promotes microglial activation-dependent neurotoxicity. This could be attenuated with the use of beta-galactosidase inhibitors, highlighting a potential target for neurodegenerative disease interventions, especially in conditions like AD and PD. Using differential gene expression analyses, An et al. identified shared crosstalk genes between AD and atherosclerosis, particularly related to complement activation and microglia signaling, emphasizing the role of immune pathways in both. They identified diagnostic genes which had over 80% predictive accuracy. They could also observe increased macrophage infiltration in both AD and atherosclerotic disease cohorts compared to healthy controls. In a single-center study, Alster et al. analyzed inflammatory markers in the cerebro-spinal fluid (CSF) and plasma of patients with supranuclear palsy, an atypical parkinsonism characterized by tauopathy, and healthy controls and correlated changes with brain volumes as examined by magnetic resonance imaging (MRI). IL-1 and IL-6 correlated variably with different brain regions, suggesting that the role of inflammatory factors in neurodegenerative disease may not be unequivocal and rather dependent on the type of inflammatory factor and selective vulnerability of certain tissues. This sentiment is echoed in Song, Li, Han, Xu, Ding et al.'s study where two-sample Mendelian randomization was employed to identify peripheral immune cells both positively and negatively associated with PD. The authors emphasize that different cell types may play different roles through the varying stages of PD and that potential therapeutic targets may be relevant at different times through the course of the pathogenesis. Similarly, Qin et al. utilized Mendelian randomization to evaluate the correlations between the plasma lipidome, circulating inflammatory proteins and risk of PD. The results show potential mediation by inflammatory proteins in the link between lipids and PD, adding to predictive markers and possible targets for PD treatment. When this method was employed by Xiao et al., to study the effects of inflammatory proteins on amyotrophic lateral sclerosis (ALS), they identified a bidirectional relationship between inflammatory markers and ALS, suggesting that ALS could also impact inflammatory protein levels, adding depth to ALS pathophysiology and potential therapeutic targets. Further examining the link between ALS and circulating inflammatory markers, Du et al. created a predictive model linking mitophagy and immune cell infiltration to ALS. They identified 40 genes with prognostic value and 4 genes with diagnostic value. They validated their differential expression in plasma from ALS patients and healthy controls. This work lays the groundwork for understanding progression of ALS and its immune and mitochondrial dynamics, potentially guiding early diagnostics and therapeutic developments.

## 4 Genetics, aging, and systemic conditions related to neurodegenerative risk

Aging is linked to altered microbiome composition, though its role in inflammation remains uncertain (Falony et al., 2016). Importantly, chronic inflammation and dysbiosis are both key features of neurodegenerative diseases and IBD. Several studies have examined the link between inflammatory bowel disease (IBD)- Crohn's disease, ulcerative colitis- and PD, yet controversy exists whether the correlation is real. Adding to this field, Wang et al. performed a large-scale cohort study and found no significant link between risk of IBD and risk of PD in general; however, specific subgroups, such as females with ulcerative colitis, did show a slight increased risk of PD. This finding provides evidence for a potential, but not universal, relationship between IBD and PD. Systemic lupus erythematosus (SLE) is an autoimmune connective tissue disease in which neuropsychiatric, particularly cognitive dysfunction, can be prevalent. Histological examination has highlighted neuronal loss resulting in loss of volume of both white and gray matter areas and elevated levels of neurofilament light protein and glial fibrillary acidic protein (GFAP) in the CSF indicating the neurodegenerative nature of these changes. Kuchcinski et al. utilized a deep learning method called BrainAGE that estimates the age gap, i.e., the difference in chronological age vs. biological age of the brain, using magnetic resonance images of patients with SLE and age matched controls. They found a higher brain age gap in SLE patients, particularly those with higher disease activity, linking SLE's inflammatory effects with accelerated brain aging and cognitive decline. There is an epidemiological association between frailty-an increased state of vulnerability- and insomnia, both of which increase with age and are risk factors for neurodegenerative diseases. Song, Li, Han, Xu, Wang et al. utilized GWAS data to determine a positive genetic correlation between the two. They identified 2 SNPs at 3p21.31 mapping to genes related to sleep regulation and inflammation. Interestingly, they found tissue specific SNP heritability enrichment with the anterior cingulate cortex and cerebral amygdala.

This editorial collectively emphasizes the interconnected nature of aging, inflammation, immune responses, and systemic factors in neurodegeneration. By synthesizing findings across these three themes, the articles provide a comprehensive view of current research, offering promising avenues for targeted interventions in aging-related neurodegenerative diseases.
